# An Aggressive Variant of Central Giant Cell Granuloma of the Posterior Maxilla With Reactive Osteogenesis: A Case Report and Review of the Literature

**DOI:** 10.7759/cureus.107261

**Published:** 2026-04-17

**Authors:** Harkanwal Preet Singh, Piyush Gandhi, Sunil MK, Bandhanjot Kaur

**Affiliations:** 1 Department of Oral Pathology and Microbiology, Dasmesh Institute of Research and Dental Sciences, Faridkot, IND; 2 Department of Oral Pathology and Microbiology, Baba Farid University of Health Sciences, Faridkot, IND; 3 Department of Oral Medicine and Radiology, Dasmesh Institute of Research and Dental Sciences, Faridkot, IND

**Keywords:** aggressive central giant cell granuloma, central giant cell granuloma, giant cells, giant cell tumours, non-odontogenic tumors, reactive osteogenesis

## Abstract

Central giant cell granuloma (CGCG) is a benign but locally destructive central lesion of the jaws with variable clinical behavior and overlapping histopathological features with other giant cell-rich lesions, posing diagnostic challenges. We report a case of a 41-year-old female patient presenting with a painless, progressively enlarging swelling in the posterior maxilla. Clinical and radiographic evaluation suggested multiple differential diagnoses. Histopathological examination revealed numerous osteoclast-like multinucleated giant cells (MGC) within a richly vascular fibrous stroma, along with areas of hemorrhage and reactive woven bone formation. Immunohistochemical analysis using p63 demonstrated negative expression, aiding in differentiation from giant cell tumor of bone and confirming the diagnosis of CGCG. The case highlights the importance of comprehensive clinicoradiological and histopathological correlation, supplemented by immunohistochemistry, for accurate diagnosis, particularly in atypical presentations. Considering the potential for aggressive behavior and recurrence, appropriate surgical management and long-term follow-up are essential for optimal outcomes.

## Introduction

Giant cell lesions of the jaws constitute a heterogeneous group of osseous pathologies encompassing entities with variable biological behavior, ranging from reactive proliferative processes to true neoplastic conditions, with occasional progression to locally aggressive or rarely malignant phenotypes [1]. The widespread occurrence of multinucleated giant cells in diverse, unrelated skeletal lesions further complicates histopathological differentiation. Secondary reactive alterations within these lesions may simulate features suggestive of malignancy, thereby necessitating advanced diagnostic expertise to accurately determine their fundamental nature [2]. Consequently, comprehensive clinicopathological correlation, incorporating radiographic assessment and, where indicated, biochemical investigations, is essential for establishing a definitive diagnosis, particularly in lesions exhibiting overlapping histomorphological characteristics within the giant cell spectrum [3].

Central giant cell granuloma (CGCG) was historically regarded as a reactive lesion; however, due to its unpredictable clinical course, potential for aggressive behavior, and its association with long bone tumors and certain syndromic conditions, it is more appropriately categorized as a benign neoplastic entity [4].

The WHO has defined CGCG as “a localized benign but sometimes aggressive, osteolytic proliferation consisting of fibrous tissue with hemorrhage and hemosiderin deposits and presence of osteoclast-like giant cells with reactive bone formation” [5]. Clinically, CGCG demonstrates a broad spectrum of biological behavior, ranging from asymptomatic, indolent, and slowly progressive lesions to aggressive forms characterized by rapid osteolysis with cortical expansion, thinning, and occasional perforation. Associated findings may include root resorption and displacement of adjacent anatomical structures such as teeth and neural elements, often accompanied by pain. Perineural spread and soft tissue infiltration are typically absent; despite the absence of a true capsule, the lesion exhibits an expansile growth pattern, displacing surrounding structures rather than demonstrating infiltrative behavior. Recurrence rates are reported to be approximately 15-20%, with a higher likelihood observed in lesions exhibiting aggressive clinical features [5].

## Case presentation

A 41-year-old female patient came with a chief complaint of swelling in the posterior maxilla. History revealed painless swelling, which was gradual in onset and slowly progressed to the present size. There was no finding of teeth loosening, difficulty in chewing, nasal obstruction, or preceding trauma. Past medical and family history were non-contributory. External examination revealed a solitary swelling on the right side of the face, resulting in obliteration of the nasolabial fold, causing facial asymmetry. A swelling of size 3 × 5 cm was located on the right side of the cheek, which was soft to firm in consistency, non-fluctuant, non-tender, with no discharge or change in overlying skin. There was no lymphadenopathy associated with it. Intraoral examination showed swelling extending from teeth 24 to 27.

**Figure 1:**
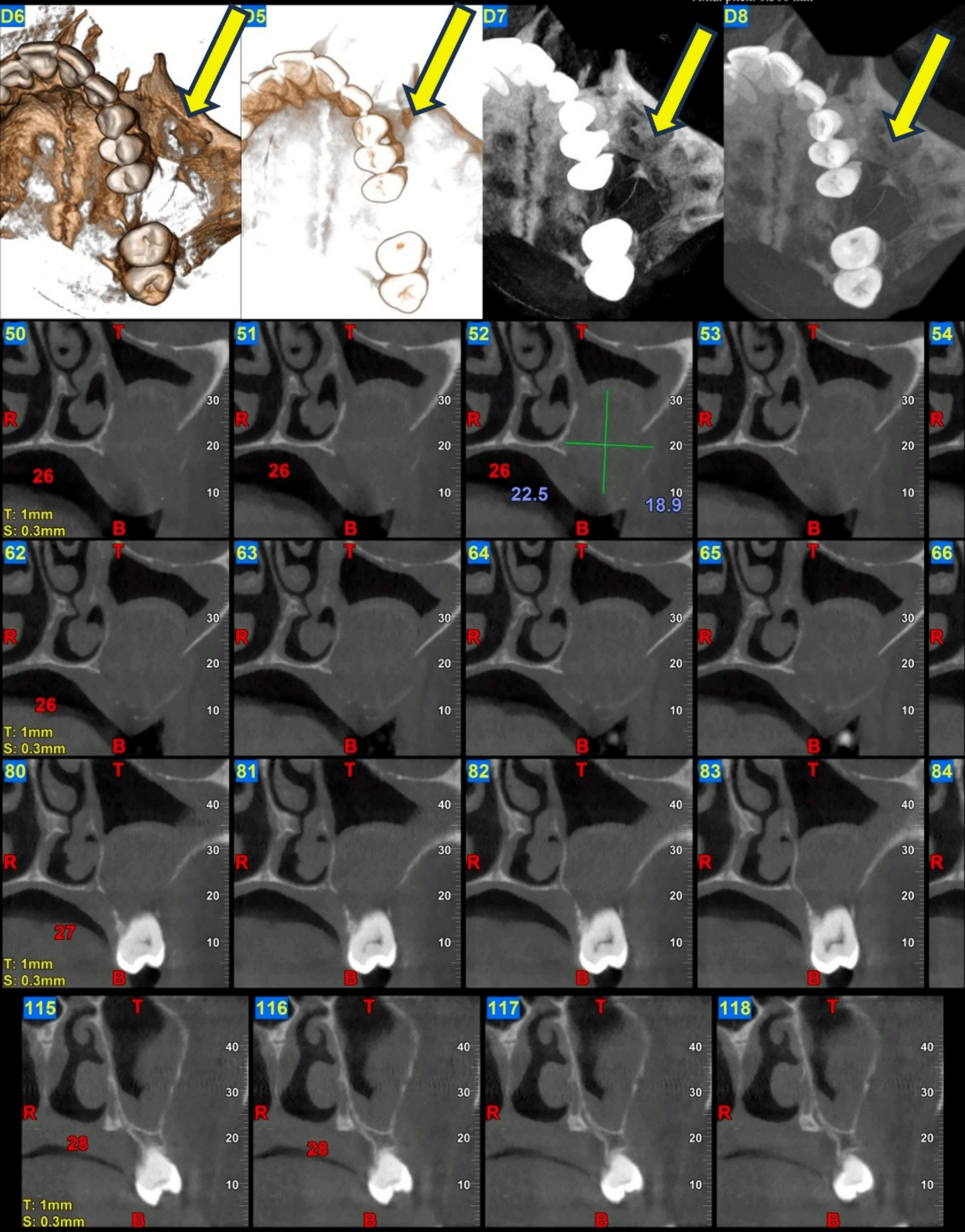
Cone-beam computed tomography (CBCT) reconstruction demonstrating a well-defined mixed radiolucent-radiopaque lesion extending from the region of teeth 23 to 27.

Cone-beam computed tomography (CBCT) reconstruction demonstrating a well-defined mixed radiolucent-radiopaque lesion involving the left hemimaxilla. The lesion extended from the region of teeth 23 to 27 and was associated with expansion of the maxillary bone, with involvement of the left maxillary sinus, and the lateral wall of the nasal cavity (Figure 1).

**Figure 2:**
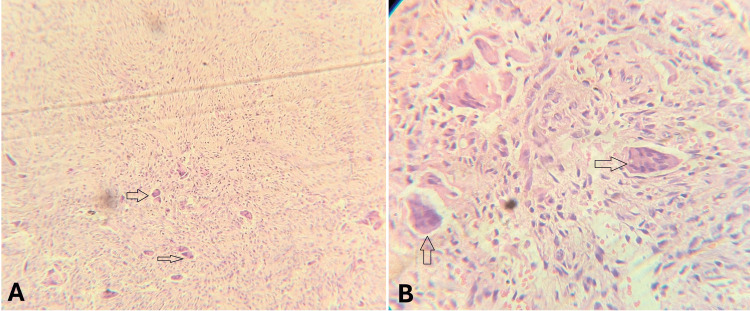
H&E-stained section showing giant cells (A: 4x; B: 40x).

Differential diagnoses included adenomatoid odontogenic tumor, odontogenic keratocyst, calcifying epithelial odontogenic cyst, nasolabial cyst, radicular cyst, giant cell tumor (GCT) of bone, and CGCG. The case was planned for surgery. Histopathologic examination revealed the presence of numerous osteoclast-like multinucleated giant cells dispersed within a highly vascularized fibrous connective tissue stroma. These giant cells contained 10 or more nuclei and were typically aggregated in focal clusters, particularly in areas associated with hemorrhage (Figure 2).

**Figure 3:**
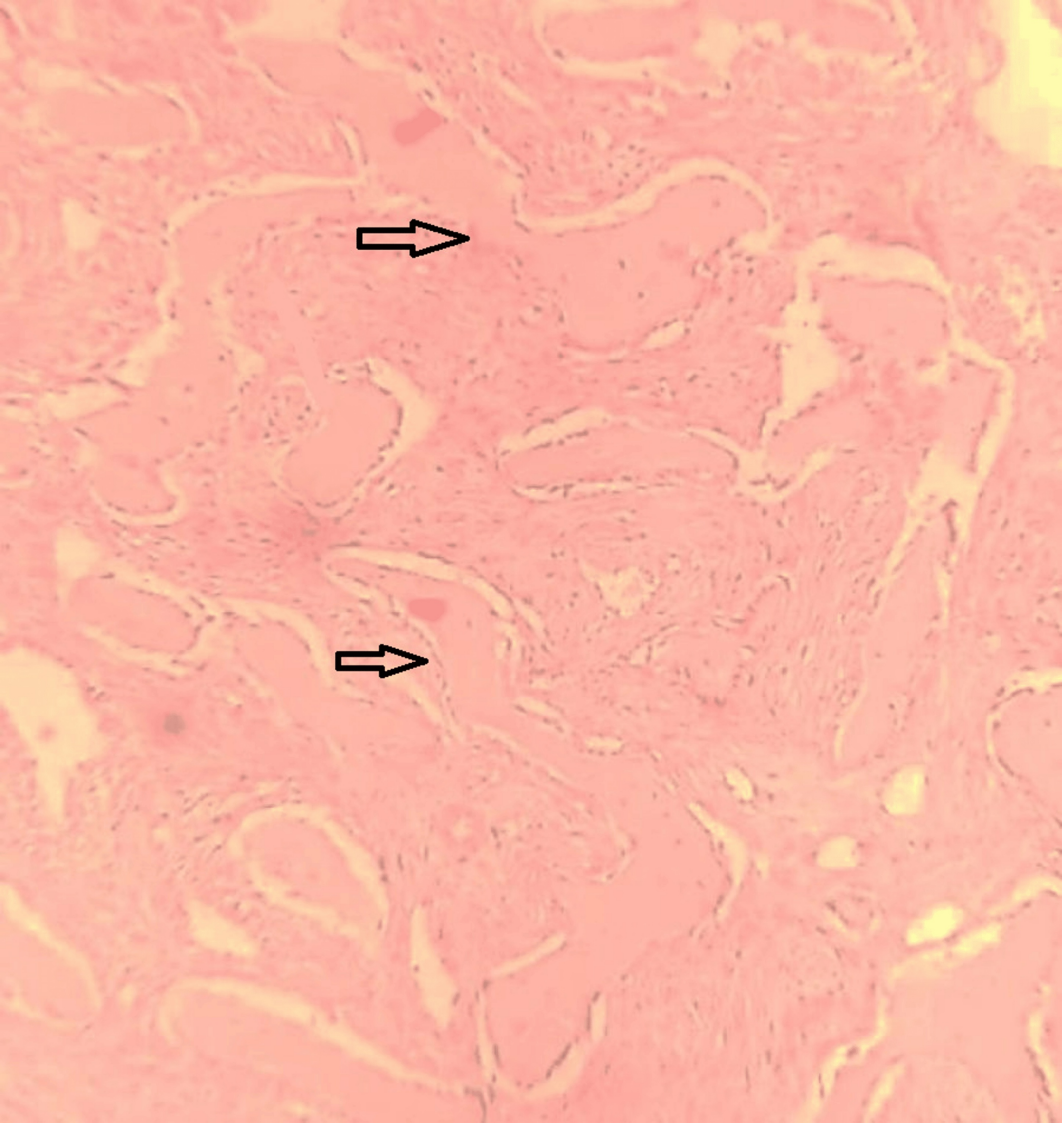
H&E-stained section showing bony trabeculae lined by osteoblasts.

The intervening stroma was composed of proliferating spindle-shaped and plump fibroblastic cells along with abundant capillary networks. Additionally, foci of reactive osteogenesis with formation of immature woven bone trabeculae were present (Figure 3).

**Figure 4:**
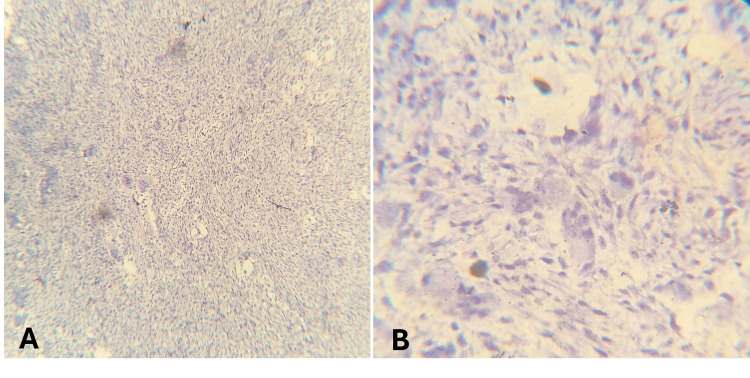
Negative immunohistochemistry expression of p63 by giant cells (A: 4x; B: 40x).

p63, a homologue of the tumor suppressor p53, located on human chromosome 3q27-29, is expressed in the mononuclear cells of GCTs of the bone, suggesting its role as a diagnostic marker. To differentiate between GCT of bone and CGCG, p63 immunohistochemistry (IHC) was used, which was negative, confirming the lesion as CGCG (Figure 4).

After the final diagnosis of an aggressive variant of CGCG of the posterior maxilla with reactive osteogenesis, surgery was planned, and enucleation with extensive curettage was done. Prognosis was good, and follow-up showed no recurrence.

## Discussion

CGCG is a benign but locally destructive and osteolytic intraosseous lesion of presumed osteoclastic lineage. Histopathologically, it is regarded as a non-neoplastic fibro-osseous process composed of a proliferative cellular connective tissue stroma containing multiple hemorrhagic foci, aggregates of osteoclast-type multinucleated giant cells, and areas of immature woven bone formation [6]. This entity predominantly affects individuals younger than 30 years, with a higher predilection for females, and constitutes approximately 7% of benign jaw lesions [7]. The etiopathogenesis remains uncertain. Earlier concepts proposed a reparative response to antecedent trauma, while alternative hypotheses suggest an underlying inflammatory mechanism. Clinically, the lesion typically presents as an asymptomatic, gradually enlarging intraosseous swelling associated with cortical expansion [8]. It is frequently detected incidentally on radiographic examination. The lesion exhibits considerable variability in size and is commonly associated with displacement or resorption of adjacent tooth roots. Radiographically, it may present as either a unilocular or multilocular radiolucent defect [9,10]. Hence, we hereby present a case of a 41-year-old patient who came with the chief complaint of swelling in the posterior maxilla and was reported as CGCG.

A 41-year-old female presented with a painless, progressively enlarging swelling in the posterior maxillary region. Provisional differential diagnoses included adenomatoid odontogenic tumor, odontogenic keratocyst, calcifying epithelial odontogenic cyst (CEOC), radicular cyst, nasolabial cyst, GCT of bone, and CGCG. Surgical excision was performed, and histopathological evaluation revealed numerous osteoclast-type multinucleated giant cells dispersed within a highly vascular fibrous stroma, containing ≥10 nuclei and arranged in focal clusters, particularly in hemorrhagic areas. The stroma consisted of proliferating spindle-shaped fibroblasts with abundant vascularity, along with areas of reactive woven bone formation. Immunohistochemical analysis using p63 was negative, thereby excluding GCT of bone and confirming the diagnosis of CGCG. Capucha et al. performed a retrospective analysis of 22 patients with 25 lesions to evaluate clinical, radiographic, and histopathological characteristics, along with treatment modalities and recurrence patterns [10]. Therapeutic outcomes were categorized as successful when regression or calcification was observed, and failure when lesions demonstrated recurrence, progression, or lack of response. The majority of patients (77%) were younger than 40 years. Lesions were more frequently located in the anterior mandible and left posterior maxilla, with common clinical manifestations including pain, dental mobility, and mucosal expansion. Radiographically, lesions were predominantly unilocular in the maxilla and multilocular in the mandible, the latter also showing a higher incidence of cortical perforation. Approximately 80% of cases were classified as aggressive. The study concluded that a combined therapeutic approach integrating both pharmacological and surgical interventions yields improved outcomes, particularly in large and aggressive lesions [10]. Chrcanovic et al. conducted a comprehensive systematic review of central giant cell lesions of the jaws, analyzing clinical and radiological characteristics with particular emphasis on factors influencing recurrence [11]. The review incorporated 365 studies encompassing 2,270 lesions. Overall recurrence was documented in 232 of 1,316 cases (17.6%), with a significantly higher rate in aggressive lesions (22.8%) compared to non-aggressive lesions (7.8%) following surgical management. Pharmacological therapy resulted in partial or complete regression in the majority of cases, although aggressive lesions exhibited a comparatively reduced response to corticosteroid treatment. Among surgically treated cases, conservative approaches such as curettage or enucleation, as well as marginal resection relative to segmental resection, were associated with increased recurrence, particularly in lesions exhibiting aggressive behavior, cortical perforation, and root resorption. Evaluation of recurrence following combined surgical and pharmacological therapy was limited due to heterogeneity in treatment protocols [11].

In the present report, to differentiate between GCT of bone and CGCG, p63 immunohistochemistry was used, which was found to be negative, confirming the lesion as CGCG. Nagar et al. evaluated p63 expression in GCT, CGCG, and peripheral giant cell granuloma (PGCG), with the aim of determining its utility as a diagnostic and differential biomarker [12]. The study included 10 cases of GCT, 20 cases of CGCG, and 20 cases of PGCG, all subjected to p63 immunostaining, with semi-quantitative assessment of positively stained cells. Statistical analysis using Kruskal-Wallis and one-way ANOVA demonstrated that p63 expression was present in 100% (10/10) of GCT cases, whereas complete absence of immunopositivity was observed in both CGCG and PGCG. This difference was statistically highly significant (p < 0.01), while no significant difference was noted between CGCG and PGCG. These findings indicate that GCT represents a distinct pathological entity and that p63 serves as a useful immunohistochemical marker for differentiating GCT from CGCG and PGCG [12].

Surgical excision is the treatment of choice, which varies from simple enucleation to resection based on the behavior of the lesion. Other non-surgical treatment modalities, such as radiotherapy, systemic calcitonin, and intralesional injection with corticosteroids, have shown promising results. After surgery, a recurrence rate of 4-20% have been reported in CGCG [13].

## Conclusions

CGCG represents a benign yet potentially aggressive intraosseous lesion with variable clinical and biological behavior, necessitating accurate diagnosis and appropriate management. The present case highlights the importance of comprehensive clinicoradiological and histopathological evaluation in establishing the diagnosis, particularly in atypical age groups and locations. Immunohistochemical analysis using p63 proved valuable in differentiating CGCG from GCT of bone, confirming its diagnostic significance. Given the risk of recurrence, especially in aggressive variants, careful treatment planning and long-term follow-up are essential.
